# Phenolic Profile and the Antioxidant, Anti-Inflammatory, and Antimicrobial Properties of Açaí (*Euterpe oleracea*) Meal: A Prospective Study

**DOI:** 10.3390/foods12010086

**Published:** 2022-12-24

**Authors:** Anna Paula de Souza Silva, Adriano Costa de Camargo, Josy Goldoni Lazarini, Marcelo Franchin, Janaina de Cassia Orlandi Sardi, Pedro Luiz Rosalen, Severino Matias de Alencar

**Affiliations:** 1 Agri-Food Industry, Food and Nutrition Department, Luiz de Queiroz College of Agriculture, University of São Paulo, ESALQ/USP, Piracicaba 13418-900, Brazil; 2 Nutrition and Food Technology Institute, University of Chile, Santiago 7830490, Chile; 3 Department of Biosciences, Piracicaba Dental School, University of Campinas, UNICAMP, Piracicaba 13414-903, Brazil; 4 School of Dentistry, Federal University of Alfenas (Unifal-MG), Alfenas 37130-001, Brazil; 5 Dental Research Division, Guarulhos University, Guarulhos 07023-070, Brazil

**Keywords:** açaí, polyphenols, antioxidant activity, NF-κB, pathogenic bacteria, agroindustrial residues, food by-products

## Abstract

The mechanical extraction of oils from Brazilian açaí (*Euterpe oleracea* Mart) produces significant amounts of a byproduct known as “meal”, which is frequently discarded in the environment as waste material. Nevertheless, plant byproducts, especially those from oil extraction, may contain residual polyphenols in their composition and be a rich source of natural bioactive compounds. In this study, the phenolic composition and in vitro biological properties of a hydroethanolic açaí meal extract were elucidated. The major compounds tentatively identified in the extract by high-resolution mass spectrometry were anthocyanins, flavones, and flavonoids. Furthermore, rhamnocitrin is reported in an açaí byproduct for the first time. The extract showed reducing power and was effective in scavenging the ABTS radical cation (820.0 µmol Trolox equivalent∙g^−1^) and peroxyl radical (975.7 µmol Trolox equivalent∙g^−1^). NF-κB activation was inhibited at 10 or 100 µg∙mL^−1^ and TNF-α levels were reduced at 100 µg∙mL^−1^. However, the antibacterial effects against ESKAPE pathogens was not promising due to the high concentration needed (1250 or 2500 µg∙mL^−1^). These findings can be related to the diverse polyphenol-rich extract composition. To conclude, the polyphenol-rich extract obtained from açaí meal showed relevant biological activities that may have great applicability in the food and nutraceutical industries.

## 1. Introduction

Agroindustrial activity generates significant amounts of solid waste throughout the processing chain [1], especially during the production of specialty oils from vegetable sources, such as açaí (*Euterpe oleracea* Mart.). This fruit belongs to the *Euterpe* gender, which comprises approximately 28 species, including *E. precatoria*, *E. edulis*, and *E*. *oleracea*. The former is also known as “açaí-do-Pará” and can be found in the Brazilian states of Pará, Tocantins, Maranhão, and Amapá, and in Guiana and Venezuela [2,3].

According to the Production of Plant Extraction and Forestry (PEVS, in Portuguese) published by The Brazilian Institute of Geography and Statistics (IBGE, in Portuguese), the açaí production in Brazil reached more than 227,000 tons, which corresponded to a sum higher than R$ 7700 million (IBGE, 2021) [4]. Açaí fruit is reported to have a high caloric value due to its lipid content (21 to 53%) [5] and to have multiple biological activities due to the presence of polyphenols, which are related to its antioxidant, anti-inflammatory, and antimicrobial properties [3,6].

Açaí oil is extracted from the pulp and has been extensively used for cosmetic and pharmaceutical applications, whereas the pulp is the main value-added commercial product for exportation. However, to extract the pulp, large amounts of residues, mainly composed of seeds and peels, are generated and frequently discarded in the environment. The Brazilian Agricultural Research Corporation (EMBRAPA) indicates that açaí seeds account for 85% of the fruit weight as compared to 15% of the pulp (epicarp and mesocarp), which can be pressed for oil extraction or consumed with other food products [7]. The mechanical processing of açaí pulp for oil extraction generates a fiber-rich byproduct named meal, which is generally discarded in the environment as a residual material. This scenario may imply several environmental damages since the yield of the byproduct is higher than its usage [8], in addition to economic losses [9].

Açaí pulp shows a bioactive potential as a source of phenolic antioxidants and other bioactive molecules [10,11,12,13,14]. Despite the fact that most studies with açaí samples investigate the edible part of the fruit [15], açaí byproducts or residues have been investigated for different purposes, such as the extraction of lignocellulosic byproducts from the residual biomass [16], the composition and antioxidant capacity of açaí seeds [8], the assessment of the antioxidant capacity and characterization of açaí fractions [15], the use of açaí pulp and seed extracts as biosorbents for residual yeasts [17], among others. However, açaí meal remains an unexplored agro-industrial byproduct with bioactive constituents, such as polyphenols, which can have potential applications as an ingredient in the food industry. 

Since it is generated from the pulp, açaí meal can be considered an important source of natural antioxidants. It showed a total phenolic content even higher than that of other byproducts, such as those generated during the *Fabaceae* processing, such as grade B soymilk powder and soy husk powder [18], methanolic and ethanolic extracts from soybean meal, and whole soybean seeds [19]. Phenolic compounds are highly effective antioxidants mainly by scavenging reactive oxygen and nitrogen species (ROS/RNS), chelating metals, and reducing free radicals. During oxidative stress, free radicals can promote deleterious effects in the organism [20,21]. Importantly, polyphenols may inhibit or mitigate oxidative and inflammatory events that are common in individuals with diabetes, obesity, cardiovascular diseases, Alzheimer’s, premature aging, and others [22,23,24,25,26]. Additionally, antioxidant compounds extracted from natural sources, such as winemaking by-products, may show the potential to act as food ingredients, preventing lipid oxidation [27] and also acting as potential replacers of synthetic antioxidants [8]. Therefore, phenolics from açaí meal may also be used as natural additives. However, there is no information available on the antioxidant, anti-inflammatory, and antimicrobial properties of açaí meal extract, which encourages further investigation for the development of novel ingredients in food and nutraceuticals. 

To the best of our knowledge, this is the first study reporting a comprehensive phenolic identification and determination of the biological activities of an extract produced from açaí meal. In this study, the phenolic profile of the dry extract of açaí meal was elucidated by high-resolution mass spectrometry (LC-ESI–QTOF-MS/MS). The extract was further tested for its antioxidant, cytotoxic, anti-inflammatory, and antimicrobial properties. This is a pioneering approach reporting the bioactive potential of açaí meal extract. 

## 2. Materials and Methods

### 2.1. Açaí Byproduct

**Figure 1:**
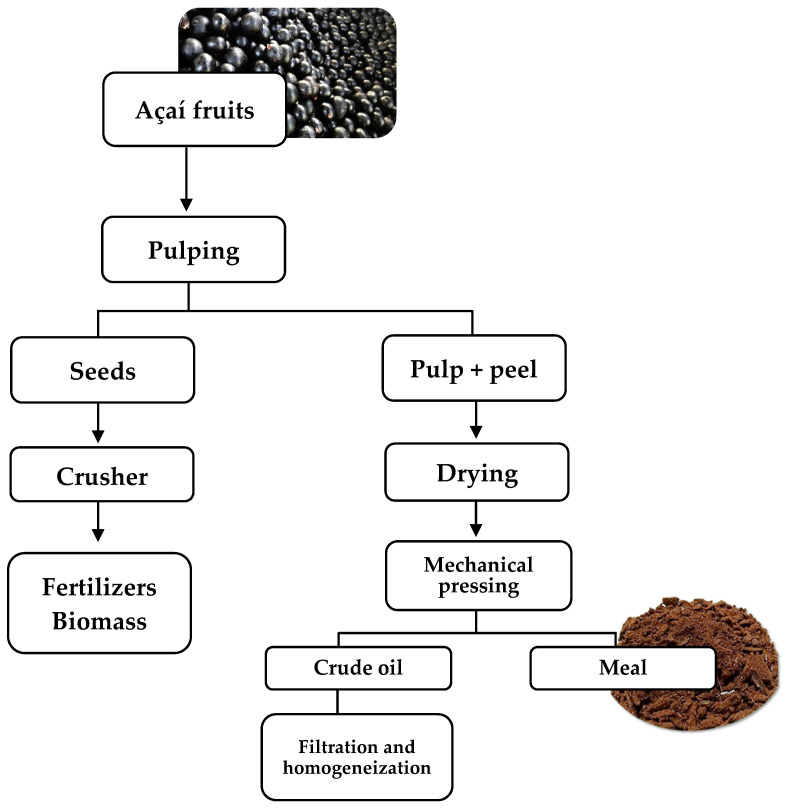
Flow diagram for the açaí processing.

Açaí meal (5 kg) samples were provided by Citróleo Industry and Commerce of Essential Oils, LTDA, Torrinha, São Paulo, Brazil. A diagram illustrating the usual extraction process of oil from açaí and the generation of açaí meal is presented in Figure 1. Notably, the meal was obtained at the end of the oil extraction from açaí pulp, which comprised the following steps: (i) Maceration of the fruits in hot water, followed by (ii) separation of pulp and seeds, (iii) drying of the pulp fraction, (iv) mechanical pressing, which yields the oil and generates the meal, and (vi) oil filtration and homogenization, recovering the açaí meal. 

After the cryogenic milling of the sample, 4 g were mixed with 20 mL of hexane (1:5, *w*/*v*) for defatting, followed by stirring, and centrifugation (5000× *g*, 10 min). This process was performed twice. The precipitate was recovered, and the solvent was evaporated. Then, the dry material was stored at −22 °C until the extract preparation.

### 2.2. Chemicals and Microbial Strains

All chemicals (reagents, standards, ELISA kit, and culture media) were procured from Sigma-Aldrich (St. Louis, MO, USA), R&D Systems (Minneapolis, MN, USA), and Promega Corporation (Madison, WI, USA). The following standard bacterial and yeast strains were used in the microbiological assays: Methicillin-resistant *Staphylococcus aureus* (MRSA, ATCC 33591), *Staphylococcus aureus* (ATCC 25923), *Klebsiella pneumoniae* (ATCC 27736), *Escherichia coli* EHEC (ATCC 43895), *Pseudomonas aeruginosa* (ATCC 27853), *Candida albicans* (MYA 2876), *C. glabrata* (ATCC 90030), *C. tropicalis* (ATCC 750), *C. parapsilosis* (ATCC 2019), *Streptococcus salivarius* (ATCC 7073), and *S. sanguinis* (SK36). The strains were grown in Sabouraud Dextrose agar (SDA, Kasvi), Brain Heart Infusion (BHI), or RPMI culture media.

### 2.3. LC-ESI-QTOF-MS/MS Phenolic Profile 

Interfering substances were removed from the extract by SPE-LC_18_, as described by de Souza Silva et al. [28]. 

Briefly, 500 mg of the freeze-dried extract from açaí meal was dissolved in purified acid water (pH 2.0). The mixture was eluted in an SPE-LC_18_ cartridge with acid water followed by methanol, which recovered the bioactive compounds from the stationary phase. Afterward, methanol was removed under N_2_ gas flow, and the remaining fraction was suspended in methanol to be injected into the LC-MS/MS system for determining the phenolic profile. 

Phenolic compounds were separated on a Phenomenex Luna C_18_ column (4.6 mm × 250 mm × 5 µm) and a Shimadzu chromatograph (Shimadzu Co, Kyoto, Japan) using the instrumental conditions described in our previous study [28]. The mobile phase was composed of A) water and formic acid (0.25%, *v*/*v*) and B) acetonitrile:water:formic acid at 80:19,75:0.25 (%, *v*/*v*). The gradient started at 10% B, increasing to 20% B (10 min), 30% B (20 min), 50% B (30 min), 50% B (32 min), 90% B (38 min), and 10% B (45 min), ending the run at 55 min. 

A Bruker Daltonics mass spectrometer (Bruker Daltonics, Bremen, Germany) equipped with an electrospray ionizer (ESI) operated with nebulizer at 2 bar, dry gas at 8L/min, the temperature at 200 °C, and 4500 V. The resolution ranged from 400 to 30,000 *m*/*z*, and data analysis was performed with the software MAXIS 3G–Bruker Daltonics 4.3 (Bruker Daltonics, Billerica, MA, USA). The tentative identification of the compounds was conducted by comparing the accurate masses from parent ion and MS2 spectra with those described in the literature. 

### 2.4. Total Phenolic Compounds, Reducing Power and Free Fadical Scavenging Capacity of Açaí Meal Extract

The freeze-dried extract was tested for its ferric-reducing antioxidant power (FRAP) and scavenging capacity against free radical ABTS and reactive oxygen species (ROS). Photometric measurements were carried out in a microplate reader (SpectraMax^®^ M3, Molecular Devices LLC, Sunnyvale, CA, USA).

#### 2.4.1. Total Phenolic Compounds (TPC) and Reducing Power

The TPC was determined following Singleton, Orthofer, and Lamuela [29], with modifications described elsewhere [28]. Briefly, 20 µL of the açaí meal extract in different concentrations, or gallic acid, were pipetted in a 96 well microplate together 100 µL of a 10% (*v*/*v*) Folin-Ciocalteu aqueous solution. The mixture reacted for 5 min in the dark and posteriorly received 75 µL of a 7.5% (*v*/*v*) sodium carbonate aqueous solution. After 40 min of reaction at room temperature in the dark, the absorbances were read at 740 nm in a microplate reader, and the results were expressed as milligrams of gallic acid equivalents (GAE) per gram of açaí meal extract (dry weight basis).

The FRAP assay was carried out as previously described by de Souza Silva et al. [28]. Briefly, 20 µL of the samples and standard (ferrous sulfate) at different concentrations were pipetted in a 96-well microplate followed by 30 μL of distilled water and 200 μL of FRAP reagent, which was composed of acetate buffer (0.3 M, pH 3.6), an iron chloride solution (20 mM) and TPTZ solution at 10:1:1 (*v*/*v*). The mixture reacted at 37 °C for 8 min and the absorbances were measured at 595 nm. The reducing power was expressed as micromoles of ferrous sulfate per gram of dry extract.

#### 2.4.2. Free Radical Scavenging Capacity

The free radical ABTS scavenging capacity and the oxygen radical absorbance capacity (ORAC) were determined according to de Souza Silva et al. [28] and Melo et al. [30], respectively. 

The ABTS radical solution was prepared by mixing the ABTS solution (7 mM) with potassium persulphate solution (140 mM), which reacted for 16 h in the dark at room temperature. The final solution was diluted with potassium phosphate buffer (75 mM, pH 7.4) to an absorbance of 0.7 ± 0.02 at 734 nm. Then, 20 µL of the samples or Trolox and 220 µL of the ABTS radical solution were pipetted in a 96-well microplate and reacted for 6 min in the dark at room temperature. After this, the absorbances were read at 734 nm, and the results were expressed as µmol Trolox equivalents per gram of dry açaí meal extract.

For the ORAC assay, aliquots of 30 µL of the samples or Trolox at different concentrations were pipetted in a 96-well microplate, followed by 60 µL of a fluorescein solution (508.25 nM) in potassium phosphate buffer (75 mM, pH 7.4), and 110 µL of AAPH solution (76 mM). The reaction occurred at 37 °C and the fluorescence was monitored every minute for 2 h. The fluorescence was monitored at 485 nm for excitation and 528 nm for emission, and the results were expressed as µmol of Trolox equivalents per gram of dry açaí meal extract. 

The extract was also evaluated for its scavenging capacity towards superoxide radical anion and hypochlorous acid (HOCl) [30]. For the superoxide radical scavenging assay, aliquots of 100 µL of the sample or standard at different concentrations, 50 µL of NBT, 50 µL of PMS, and 100 µL of NADH were sequentially pipetted in a 96-well microplate. The absorbance was monitored at 560 nm every 1 min for 5 min of reaction, and the results were expressed as EC_50_, that is, the concentration (µg mL^−1^) of the dry extract capable of quenching 50% of superoxide radicals [30]. For the HOCl scavenging assay, aliquots of 100 µL of potassium phosphate buffer (100 mM, pH 7.4), 100 µL of different concentrations of the sample or standard, 50 µL of dihydrorhodamin 123 (DHR, 7.5 µM), and 50 µL of a HOCl solution (30 µM) were pipetted sequentially in a 96 well microplate. The microplate was incubated at 37 °C and fluorescence was read at 528 ± 20 nm for emission and 485 ± 20 nm for excitation, and the results were also expressed in EC_50_.

### 2.5. In Vitro Cytotoxicity and Anti-Inflammatory Activity

#### 2.5.1. Cell Culture

The anti-inflammatory effects of the extract were determined according to our previous study [28]. RAW 264.7 macrophages (ATCC^®^ TIB-71™) transfected with the NF-κBpLUC gene (CQB N° 022/97) were cultured in RPMI media supplemented with fetal bovine serum (10%), penicillin (100 U/mL), and glutamine (2 mM). Cells were grown at 37 °C under 5% CO_2_.

#### 2.5.2. Cell Viability Assay

The MTT assay was performed to measure cell viability upon exposure to different concentrations of the açaí meal extract. Briefly, macrophages were seeded (2 × 10^5^ cells∙mL^−1^) onto 96-well plates and incubated at 37 °C in 5% CO_2_ for 24 h. Next, cells were treated with the extract at 1, 10, or 100 µg∙mL^−1^ and incubated for 24 h. The supernatant was discarded and an MTT solution (0.3 mg∙mL^−1^ in RPMI) was added to each well. The plate was incubated for 3 h, then the supernatant was removed, and 200 µL of DMSO was pipetted into the wells. The absorbance of the wells was measured at 540 nm in a microplate reader (SpectraMax M3, Molecular Devices, LLC, Sunnyvale, CA, USA). 

#### 2.5.3. NF-κB Activation and TNF-α Quantification

Macrophages were seeded onto a 24-well plate (3 × 10^5^ cells per well) and incubated under the same conditions described earlier. Next, cells were treated with the extract at 1, 10, or 100 µg∙mL^−1^ for 30 min and then stimulated with LPS (10 ng∙mL^−1^) for 4 h. The supernatant was recovered and TNF-α levels were quantified by ELISA in a microplate reader following the manufacturer’s instructions. The results were expressed as pg∙mL^−1^. 

To determine the effects of the extract on NF-κB activation, once the supernatant was recovered, macrophages were lysed (TNT lysis buffer) and 10 µL of the suspension was mixed with 25 µL of Luciferase (a reagent containing luciferin). Luminescence was quantified in a microplate reader (SpectraMax^®^ M3, Molecular Devices LLC, Sunnyvale, CA, USA).

### 2.6. Antimicrobial Activity of the Açaí Meal Extract

#### 2.6.1. Strains and Growth Conditions

Bacteria and yeast strains of medical-dental and/or food interest were maintained as frozen stocks at −80 °C until used. After thawing, they were inoculated in BHI broth and incubated at 37 °C for 24 h. Bacterial and yeast inocula were prepared in sterile saline solution (0.9 %) to 5 × 10^8^ CFU∙mL^−1^ and 2.5 × 10^6^ CFU∙mL^−1^, respectively. The suspensions were diluted in Müeller-Hinton (bacteria) or RPMI (yeast) media to obtain an approximate final concentration of 10^5^ CFU∙mL^−1^ for bacteria and 10^4^ CFU∙mL^−1^ for yeasts.

#### 2.6.2. Minimum Inhibitory Concentration (MIC)

The MIC of the extract was determined by the microdilution method according to the Clinical and Laboratory Standards Institute M27-S3 [31] and M07-A9 [32]. The açaí meal extract was mixed with sterile saline solution (0.9%) at 30 mg∙mL^−1^. After solubilization, 100 μL of the samples were added to the wells and serially diluted to obtain concentrations ranging from 9.76 to 5000 μg∙mL^−1^. Then, aliquots of the microbial suspensions (100 μL) were added to the wells and the microplates were incubated at 37 °C for 24 h under appropriate growth conditions. 

After incubation, 50 µL of resazurin (0.01%) was added to each well and the microplate was incubated again at 37 °C for 2 h. The MIC was defined as the lowest concentration of the extract that prevented the growth-induced color change promoted by resazurin. Color change from blue (original resazurin color) to pink indicates the presence of viable cells. Standard antimicrobials were used as positive controls and inoculated culture media served as a negative control. All assays were performed in triplicate of three independent experiments.

#### 2.6.3. Minimum Bactericidal and Fungicidal Concentration (MBC/MFC)

To determine the MBC/MFC, 10 µL of the wells corresponding to the MIC and higher concentrations were subcultured onto BHI agar or SDA plates. The plates were incubated at 37 °C for 24 h. The MBC/MFC was defined as the lowest concentration of açaí meal extract that prevented visible microbial growth on the solid media.

### 2.7. Statistical Analysis 

The assessments were made in triplicate and results were expressed as mean ± standard deviation. Tukey’s post-test was performed for the antioxidant capacity, cytotoxic and anti-inflammatory activities. In all statistical tests, the significance level was considered at *p* < 0.05.

## 3. Results and Discussion

### 3.1. Comprehensive Chemical Characterization of the Extract from Açaí Meal 

The açaí meal extract was subjected to LC-ESI-QTOF-MS/MS analysis in the positive mode for the identification of anthocyanins, and in the negative mode for the identification of flavonoids and non-flavonoids (Table 1).

Cyanidin 3-*O*-glucoside and cyanidin 3-rutinoside showed a fragment at *m*/*z* 287, which is also typical of their aglycone forms [33]. Cyanidin 3-*O*-glucoside was previously identified in grape peels, and when isolated, it was able to inhibit mammal tumor cells [34]. This is one of the many anthocyanin monomers spread in nature [35], which is reported to have anti-inflammatory activity by inhibiting lipopolysaccharide (LPS)-induced activation of the nuclear factor kappa B (NF-κB), among other mechanisms [35,36]. Delphinidin 3-rutinoside exhibited an *m*/*z* signal at 611.1606 and was tentatively identified in the açaí meal extract at *m*/*z* 303.0505, which is characteristic of a loss of 308 Da (162 + 146). This can be related to the link of a hexose as well as a deoxyhexose at the same position as the aglycone form [37].

Pelargonidin 3-*O*-glucoside showed a protonated ion at *m*/*z* 433.1136 and a fragment at *m*/*z* 271.0607, due to the loss of a hexose [M+H-162]^+^ [38]. According to Zhang et al. [39], pelargonidin 3-glucoside isolated from strawberry extracts showed antiproliferative activity on oral, cervical, prostate, and colon cells. Peonidin 3-*O*-rutinoside also showed the same behavior as delphinidin 3-rutinoside, losing 308 Da and generating a fragment ion at *m*/*z* 301.0725 [37]. 

Peonidin 3-*O*-glucoside showed a [M+H]^+^ at *m*/*z* 463.1243 and an MS/MS fragment at *m*/*z* 301, which corresponds to the aglycone form of this anthocyanin, as a result of the loss of one hexose [M+H-162]^+^. Malvidin 3-glucoside (*m*/*z* at 493.1346) showed an MS^2^ fragment at *m*/*z* 331.082. The loss of 162 Da corresponds to a neutral loss of a glucoside group [40].

In general, anthocyanins are reported to have multiple biological activities, such as antioxidant and anti-inflammatory [41,42], with potential beneficial health effects, such as in the prevention of cognitive dysfunction and Alzheimer’s disease [43,44]. The antimicrobial potential of anthocyanins, such as cyanidin 3-*O*-glucoside, against important foodborne pathogens (*Escherichia coli* and *Staphylococcus aureus*) has also been investigated. The results indicate that the complex cyanidin 3-*O*-glucoside plus lauric acid could be adopted mainly as a food preservative or even as a therapeutic ingredient against the aforementioned bacteria strains [45].

Caffeoylquinic acid (deprotonated ion at *m*/*z* 353.0890) and 4-caffeoylshikimic acid (deprotonated ion at *m*/*z* 335.0722) were the only phenolic acids identified in the extract. Caffeoylquinic acid belongs to the hydroxycinnamic acids group and is an ester of caffeic and quinic acids. This compound is mainly found in coffee beans and beverages [46] and was beneficial in the management of metabolic syndrome, with antidiabetic, anti-inflammatory, and antioxidant potential [47]. The fragmentation of caffeoylquinic acid generated *m*/*z* signals at 191.0562, 179.034 [48], and others [49]. 

The compound 4-caffeoylshikimic acid is an isomer of caffeoylshikimic acid and was previously identified in *Euterpe oleracea* root extracts [50]. This compound showed MS/MS fragments at *m*/*z* 179.0347, 161.0319, and 135.0464, which is in line with the fragmentation pattern reported by Brunschwig et al. [50]. Generally, hydroxycinnamic acids have multiple biological activities, mainly antioxidant, anti-inflammatory, and antimicrobial.

**Figure 2:**
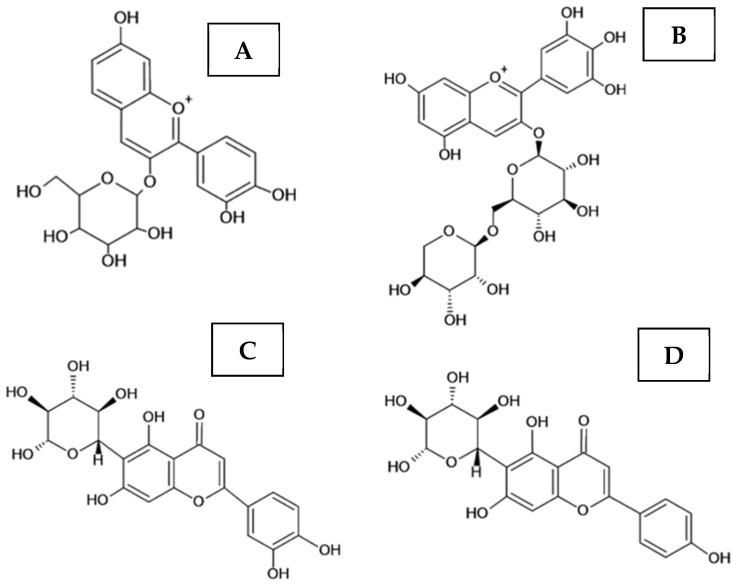
The structural formula of the main flavonoids identified in the açaí meal extract (Table 1). (**A**) = Cyanidin 3-*O*-glucoside, (**B**) = Delphinidin 3-rutinoside, (**C**) = 6-C-glycosyl luteolin (isoorientin), (**D**) = 6-C-glycosyl apigenin (isovitexin).

Isoorientin (Figure 2) and vicenin-2 were previously shown to have anti-inflammatory activity. 

Likewise, isoorientin exhibited antibacterial effect against *Bacillus subtilis* ATCC 11562 [51]. Vicenin-2 (6,8-di-C-hexosyl apigenin), a deprotonated ion at 593.1503, was identified in the açaí meal extract. Vicenin-2 is formed by the presence of aglycone apigenin (270) and 2 units of hexoses (162 + 162), with a final molecular weight of 594 Da [52]. Isovitexin, another apigenin derivative (Figure 2), was also found in the extract. This compound showed a deprotonated ion at *m*/*z* 431.0975, followed by *m*/*z* 311 and 341, which corresponds to losses of 120 and 90 Da [52]. 

Isovitexin showed anti-inflammatory activity by selectively inhibiting COX-2 and NO production in LPS-stimulated RAW cells [53] and downregulating the production of pro-inflammatory mediators [54]. Other authors pointed out that luteolin glycosides demonstrate low anti-inflammatory activity in vitro but high activity in vivo since they are transformed into glucuronides that are selectively activated in inflamed sites [55]. 

Three luteolin derivatives were tentatively identified as isoorientin isomers but they might be orientin isomers, which have the same fragmentation pattern [50,56]. A trend in the fragmentation of the deprotonated ions at *m*/*z* 447.09 can be observed, while the losses of 90 Da correspond to 6-C-glucosylation. In addition, the fragments *m*/*z* 327 and 357 in the three identified isoorientins are strongly related to glycosylated luteolin derivatives [50,52].

To the best of our knowledge, this is the first time that rhamnocitrin is reported in an açaí product. This compound was previously detected in *Rhamnus prinoides* with deprotonated ion at *m*/*z* 299 [57]. Both its parent ion and MS/MS fragments (*m*/*z* 299 and 284) are consistent with the findings of this study (Table 1). Rhamnocitrin is a flavonoid derived from kaempferol with reported strong antioxidant and anti-inflammatory activities, also exerting antimicrobial activity against *S. aureus* at 50 μg∙mL^−1^ [58]. Other biological properties have been attributed to this compound. Rhamnocitrin isolated from *Bauhinia variegata* stem bark presented anticataract activity that is possibly linked to its strong antioxidant capacity and effectiveness against the progression of damages promoted by H_2_O_2_ and GSH depletion in lenses [59]. Aglycones and glycoside forms of rhamnocitrin were also identified in green fruits of *Rhamnus* species [60].

Taxifolin-3-*O*-glucoside showed a deprotonated ion at 465.1025 and was tentatively identified according to its MS^2^ fragmentation at *m*/*z* 285.0409, 151.0035, and 125.0728 [61]. The fragment signal at m/z 285 indicates the loss of glucose and water (162 + 18) [60].

Rutin ([M-H]^−^ at *m*/*z* 609.1435) is a quercetin derivative mainly found in buckwheat, apricots, grapes, and some citric fruits. This glycosylated flavonoid has multiple biological activities, mainly antioxidant and anti-inflammatory [62]. Rutin was reported to inhibit tumor cell proliferation and protect healthy cells from oxidative stress, DNA damage, and inflammatory processes by decreasing ROS production [63]. Rutin fragmentation is characterized by the loss of 308 Da, which is a typical fragment of rutinoside [64].

The flavanone eriodictyol-7-O-glucoside I, [M-H]^−^ at *m*/*z* 449.1079, was identified in the açaí meal extract and is characterized by the loss of a hexose [M-H-162]^−^. This flavonoid was already identified in açaí juice [65]. Eriodictyol showed antimicrobial [66] and anti-inflammatory properties by inhibiting the synthesis of proinflammatory cytokines [67].

Lastly, the flavonol scoparin was tentatively identified with a deprotonated ion at 461.10 and MS^2^ fragments at *m*/*z* 341 and 371, which corresponds to losses of 120 and 90 Da. This compound was previously found in an açaí dietary supplement [68].

Collectively, the LC-ESI-QTOF-MS data and previous literature reports suggest that the phenolic compounds tentatively identified in the açaí meal extract are likely to scavenge free radicals and possess anti-inflammatory and antimicrobial activity. Açaí meal still contains several residual phenolic compounds that may present these properties, and upon recovery, could be used in the food and health industry.

### 3.2. Antioxidant Capacity: TPC, Reducing Power and Free Radical Scavenging 

Several studies indicate that total phenolic compounds (TPC) recovered from plant materials can be correlated with antiradical activity, reducing power and the protective effect against ROS-induced DNA damage [69,70,71,72]. The TPC and scavenging effects of the açaí meal extract on the synthetic radical ABTS radical cation, ion reduction (FRAP), and ROS deactivation (peroxyl radical, superoxide anion, and hypochlorous acid) are shown in Table 2.

Regarding the TPC, the result of this work (Table 2) was higher than that determined by Kang et al. (2012) [73] for *E. oleracea* pulp extract (31.2 mg GAE∙g^−1^), for methanolic extracts of Colombian açaí analyzed by Garzón et al. (2017) (47.86 mg GAE∙g^−1^) [33], and for açaí seeds extract analyzed by Melo et al. (2021) (64.58 ± 1.89 mg GA∙g^−1^) [8]. Açaí meal extract also presented higher TPC than the açaí freeze-dried pulp analyzed by Batista et al. (2016) [74], which found 55.20 mg GAE∙g^−1^ for the extract obtained without supercritical extraction, and values ranging from 54.57 a 75.65 mg GAE∙g^−1^ when the supercritical extraction was applied. Additionally, açaí meal extract showed superior reducing power assessed by TPC assay than different waste from wine and cider industries, namely grape marc (11.40 ± 0.23 mg GAE∙g^−1^), grape stalks (15.37 ± 0.31 mg GAE∙g^−1^) and apple pomace (6.5 mg GAE∙g^−1^) [75].

The phenolic profile and antioxidant properties of methanol extracts from Colombian açaí were investigated by Garzón et al. [33]. The samples scavenged the ABTS^•+^ radical at 210.49 ± 30.71 µmol Trolox∙g^−1^, while the açaí meal extract tested in this study showed a four-fold higher activity (820.0 ± 36.4 µmol Trolox∙g^−1^) (Table 2). The extraction method and sample concentration can significantly influence the sample effectiveness [76]. The açaí meal extract also showed an ABTS radical scavenging capacity superior to three byproducts of wine grapes cultivars: Chenin Blanc (218 µmol Trolox∙g^−1^), Petit Verdot (626 µmol Trolox∙g^−1^), and Syrah (653 µmol Trolox∙g^−1^) [30].

Likewise, the açaí meal extract exhibited greater ABTS radical cation scavenging capacity than the extract obtained from açaí seeds (763.09  ±  17.27 µmol Trolox∙g^−1^) [8]. The açaí meal extract also showed higher ABTS radical cation scavenging activity than ethanolic extracts obtained from avocado by-products (cultivars Hass and Fuerte), exhibiting 1.2- and 1.4-times higher antiradical activity than Hass and Fuerte seeds extracts, respectively [77].

When compared to optimized ethanolic extract from açaí fruit [78], açaí meal extract also showed a superior capacity to scavenge ABTS radical cation, showing a result about 54 times higher. The extract from açaí meal also showed a higher capacity of scavenging against ABTS radical cation when compared to optimized extracts from açaí juçara fruits (*Euterpe edulis* Mart.) [79]. Extracts of açaí juçara pulp obtained with water, ethanol 99.9%, and ethanol 70% showed antioxidant activity of 16.53 ± 0.20, 15.96 ± 0.07, and 17.52 ± 0.01 µmol Trolox∙g^−1^ against ABTS radical cation, lower values compared to the açaí meal extract. However, the behavior presented by Silva et al. [79] is in accordance with the trend observed in this study regarding the polarity of the extractor solvent, indicating that a moderate-polar solvent can be more efficient than pure ethanol for the antioxidant activity. As shown in Table 2, the extract showed reducing power at 986.0 ± 22.0 µmol FS∙g^−1^. These values are higher than those of other residues from the pulp, raw peel, seeds, lyophilized peel, and oven-dried peel of different fruits, such as avocado, pineapple, banana, and watermelon [80]. 

Since the presence of phenolic compounds in a sample can be correlated to its antioxidant activity [81], a correlation analysis was performed for TPC and antioxidant activity assessed by FRAP and ABTS radical scavenging assays of the sample tested in this study. TPC and FRAP showed a positive and strong correlation with R = 0.8531, and TPC and ABTS radical scavenging assay showed a positive and very strong correlation with an R = 0.9730. Hence, this statistical analysis indicate that açaí meal extract is a material with high antiradical activity which is correlated to the phenolic content, showing a diverse profile in polyphenols as presented in Table 1. Additionally, considering that the processes of free radical generation are involved in the activation of NF-κB, it would be possible to suggest that the anti-inflammatory activity is due to, at least in part, the antioxidant properties of the phenolic compounds present in the extract.

The antioxidant activity of pulps from *Euterpe precatoria Mart.*, known as *açaí-do-Amazonas*, and *E. oleracea Mart.*, *açaí-do-Pará*, were evaluated by Kang et al. [73]. The same authors reported that the peroxyl radical scavenging activity of *açaí-do-Amazonas* (1828.4 µmol Trolox∙g^−1^ of dry extract) was stronger than that of *açaí-do-Pará* (1014.0 µmol Trolox∙g^−1^ of the dry extract [73]. This is consistent with the ORAC data showing that the extract from *açaí-do-Pará* meal scavenged the peroxyl radical at 975.7 ± 69.0 µmol Trolox∙g^−1^ of dry extract (Table 2). These findings suggest that the meal extract had a peroxyl radical scavenging activity similar to that of the pulp extract. 

**Figure 3:**
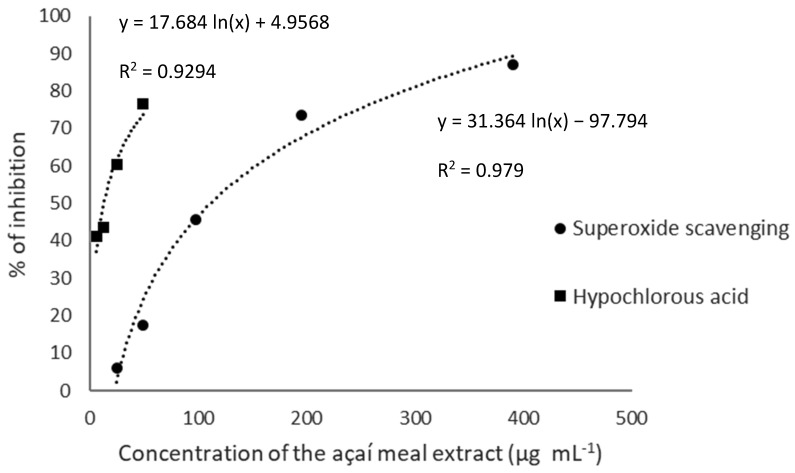
EC_50_ graphics showing the scavenging of superoxide and hypochlorous acid of the optimized açaí meal extract. The figure indicates the scavenging percentage (%) as a function of the extract concentration (µg∙mL^−1^).

The açaí meal extract showed a stronger peroxyl scavenging capacity (Figure 3) than that of Brazilian native plants studied by Infante et al. [82], that is, 4- and 1.5-fold higher than that of *Eugenia neonitida* Sobral extract and its phenolic-rich fraction, respectively [83]. 

Superoxide anion (O_2_^•−^) production by the electron transport chain occurs spontaneously and may increase during the inflammatory process. Superoxide anion can be converted into hydrogen peroxide by the superoxide dismutase enzyme, which can interact with transition metals and produce hydroxyl radical—the starter of the lipid peroxidation chain and a precursor of peroxyl radical [21]. The açaí meal extract showed a higher capacity to scavenge superoxide anion when compared to extracts from the seeds of *Eugenia brasiliensis*, *E. involucrate*, and *E. myrcianthes* [83].

Hypochlorous acid (HOCl) is a highly oxidative species generated inside neutrophils in response to microbial attacks. HOCl is generated during the oxidation of chloride ions by hydrogen peroxide through the action of myeloperoxidase. When it reaches high concentrations in the organism, HOCl may be sharply damaging [20]. The açaí meal extract was found to effectively scavenge HOCl, with an EC_50_ of 4.2 ± 0.7 µg∙mL^−1^ (Figure 3). Ethanolic extracts obtained from avocado by-products (peels and seeds) were capable to quench 50% of HOCl at concentrations ranging from 5.2 to 8.6 µg∙mL^−1^ [77]. The EC_50_ concentrations of extracts produced from rachis and pomaces of different grape cultivars ranged from 17 to 128 µg∙mL^−1^ [30], being the açaí meal extract also 1.18 to 7.4-times higher in the quench of HOCl than some extracts obtained from Brazilian native fruits [84], and 8.3 times higher even when compared to white açaí juice [85]. Additionally, the açaí byproduct extract showed higher activity in quenching HOCl when compared to Trolox (134. µg∙mL^−1^) [86]. Therefore, the present results indicate that the açaí meal extract can be more effective in quenching hypochlorous acid rather than extracts obtained from other plant byproducts or fruits.

### 3.3. In Vitro Cytotoxicity and Anti-Inflammatory Activity

**Figure 4:**
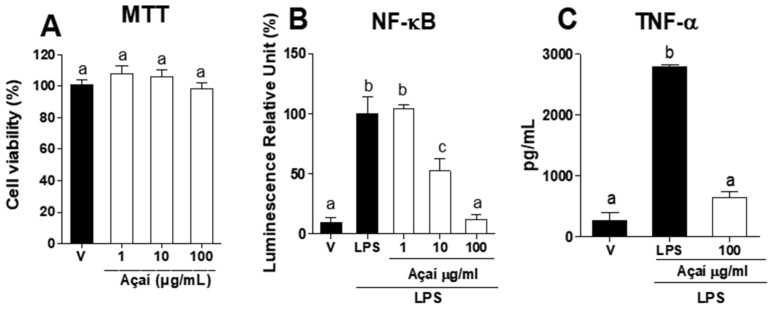
The inhibitory activity of the optimized açaí meal extract on NF-κB activation and TNF-α levels. (**A**) Viability of macrophages exposed to the extract at 1, 10, or 100 μg∙mL^−1^. (**B**) NF-κB activation in cells pretreated with the extract at 1, 10, or 100 μg∙mL^−1^ after stimulation with lipopolysaccharide (LPS) (10 ng∙mL^−1^) for 4 h. (**C**) TNF-α levels detected in the supernatant of cultures pretreated with the extract at 100 μg∙mL^−1^ after stimulation with LPS (10 ng∙mL^−1^) for 4 h. The data were expressed as mean ± SEM, *n* = 4. V—vehicle. Different letters indicate significant statistical differences (*p* < 0.05).

The açaí meal extract was further tested for its cytotoxic effects on macrophages and anti-inflammatory activity *in vitro*. Cells treated with the extract at 1, 10, or 100 µg∙mL^−1^ showed 100% viability, with no significant difference compared to the vehicle control (*p* > 0.05) (Figure 4A).

Sprenger et al. [87] found that the hydroethanolic extracts of açaí leaves, pulp, and seeds were nontoxic to treated cells, with LC_50_ of 2033, 1053, and 1310 µg∙mL^−1^, respectively. Dias-Souza et al. [88] evaluated the cytotoxicity of an açaí pulp extract produced with 80% (*v*/*v*) methanol against liver carcinoma cells. The authors reported that treatment with the extract at 15.62 µg∙mL^−1^ decreased tumor cell proliferation and did not affect the viability of cells used as controls, which is consistent with the viability data of the present study.

The inflammatory process is a natural response mechanism against the invasion of microorganisms and other antigens into the body tissues or bloodstream, as well as in cell death, tissue damage, and diseases such as cancer [89,90]. In this study, the açaí meal extract was tested for its ability to modulate the activation of the NF-κB transcription factor (Figure 4B) and TNF-α release (Figure 4C) in LPS-stimulated cells at nontoxic concentrations. The data demonstrated that treatment with the extract at 10 or 100 μg∙mL^−1^ significantly reduced NF-κB activation compared to the LPS-control group (*p* < 0.05). Interestingly, NF-κB activation levels between cells treated with the extract at 100 μg∙mL^−1^ and the control (V) were not statistically different, highlighting the potency of the phenolics present in the extract. Additionally, the group treated with the extract (100 μg∙mL^−1^) showed significantly lower TNF-α levels compared to the LPS control (*p* < 0.05).

Ethyl acetate extracts of the açaí species *E. precatoria* and *E. oleracea* were tested for their anti-inflammatory properties by Kang et al. [73]. The authors reported that *E. precatoria* extract at 20 µg_∙_mL^−1^ inhibited LPS-induced NF-κB activation by 23%, whereas *E. oleracea* extracts did not show any activity. A strong inhibition can be observed regarding the NF-κB factor and TNF-α levels in cells treated with the meal extract obtained from *E. oleracea* processing. 

Previous studies indicated that the extracts from the pulp of açaí and the juice can inhibit NF-κB activation and TNF-α synthesis. Taken altogether, these findings suggest that different parts of açaí could be used to prevent the onset and/or aggravation of inflammatory conditions such as atherosclerosis [6,91,92]. More importantly, açaí byproducts may be a new source of compounds with strong anti-inflammatory potential to be further explored for the development of drugs, cosmetic formulations, and/or even to be incorporated into functional foods.

### 3.4. Antimicrobial Activity

As shown in Table 3, the açaí meal extract showed antimicrobial activity against *S. aureus*, *MRSA* and *P. aeruginosa*, with MIC/MBC values (µg∙mL^−1^) of 1250/1250; 2500/2500, and 2500/2500, respectively. 

Other relevant pathogenic strains [*Escherichia coli* EHEC (ATCC 43895), *Candida albicans* (MYA 2876), *C. glabrata* (ATCC 90030), *C. tropicalis* (ATCC 750), *C. parapsilosis* (ATCC 2019), *Streptococcus salivarius* (ATCC 7073), *S. sanguinis* (SK36) and *Klebsiella pneumoniae* (ATCC 27736)] were also tested, however the extract did not show any significant antimicrobial activity against them.

Phenolic compounds such as flavan-3-ols, flavonols, and anthocyanins are known to have antimicrobial activity stronger than that of other polyphenols. Compounds belonging to these groups were identified by LC-MS in the optimized açaí meal extract. Their mechanisms o.f action are diverse, including alterations in cell membrane permeability, inhibition of biofilm formation, neutralization of bacterial toxins, and, importantly, the ability to interact synergistically with antibiotics and/or other natural antimicrobial substances [93,94].

The antimicrobial property of phenolic compounds can be influenced by their solubility and affinity of functional groups with the microbial cell membrane. For instance, the hydroxyl group can interact with components of the membrane and promote its disruption with consequent extravasation of the intracellular content [95]. Moreover, the position of the hydroxyl group in the molecules can also influence their biological effects by affecting the charge gradient and potential difference of the membrane. Through this mechanism, the adenosine triphosphate pool is drastically reduced leading to cell death [96]. 

According to Newman and Cragg (2020) [97], 64% of new drugs discovered and approved by the FDA (Food and Drug Administration) were natural products or derivatives thereof. These data not only strengthen scientific research in this sector, but also increase the challenges for researchers to identify new molecules, elucidate the control of action and propose therapeutic use. However, the MIC/MBC values (µg/mL) for *S. aureus*, *S. aureus* MRSA and *P. aeruginosa* were, respectively, 1.250/1.250, 2.500/2.500 and 2.500/2.500. Studies carried out by Freires et al. (2015) [98] demonstrated that MIC at concentrations from 1000 to 2000 have weak antibacterial activity and above this value is considered without activity. Thus, the authors decided not to invest in other more complex tests. However, the reporting of these results is relevant since this is the first study exploring the antimicrobial activity of an açaí meal extract.

## 4. Conclusions

The findings of this study demonstrated that the açaí meal byproduct is a rich source of phenolics compounds with multiple in vitro biological activities, mainly antioxidant and anti-inflammatory. The chemical characterization of the açaí meal extract showed a diverse phenolic composition. A positive and strong correlation was found between total phenolic content and antiradical activity for FRAP and ABTS radical scavenging assays. This is the first time that the anti-inflammatory potential of açaí meal is assessed, with important results regarding the inhibitory effects of NF-κB activation and TNF-α release. The açaí meal did not show a promising potential for antimicrobial activity against bacteria belonging to the ESKAPE group. 

Therefore, açaí meal could be considered a new natural source of antioxidant compounds with related biological activities for potential industrial applications. This work opens new possibilities for studies to investigate the effects of the açaí meal extract on lipid matrices as a natural antioxidant. By assessing the biological potential of açaí meal and determining its phenolic composition, the present study may impact the açaí production chain. Açaí meal is mostly discarded in the environment, but as shown here, it has great potential to become a value-added product. In summary, açaí meal could have great applicability in the food, pharmaceutical, and cosmetic industries. Future studies, should consider new lots and possible variations due to crop year/seasonality. Likewise, the findings observed in our study require validation in food systems or animal/human trials depending on the final application. 

## Figures and Tables

**Table 1:** Phenolic compounds tentatively identified in the optimized açaí meal extract by LC-ESI-QTOF-MS/MS.

	Compounds	Retention Time (min)	Molecular Formula	Exact Mass	Parent Ion (*m*/*z*)	Error (ppm)	Fragments (MS^2^)
Anthocyanins [M+H]^+^	Cyanidin 3-*O*-rutinoside	11.4	C_27_H_30_O_15_	594.1574	595.1652	−1.346	287.0556
	Cyanidin 3-*O*-glucoside	16.9	C_21_H_20_O_11_	448.1018	449.1096	−13.836	287.0548
	Delphinidin 3-rutinoside	19.0	C_27_H_30_O_16_	610.1528	611.1606	−0.656	303.0505
	Pelargonidin 3-*O*-glucoside	19.3	C_21_H_20_O_10_	432.1058	433.1136	1.389	271.0607
	Peonidin 3-*O*-rutinoside	19.7	C_28_H_32_O_15_	608.1736	609.1814	−0.987	301.0725
	Peonidin 3-*O*-glucoside	23.1	C_22_H_22_O_11_	462.1165	463.1243	0.649	301.0722
	Malvidin 3-glucoside	23.1	C_23_H_24_O_12_	492.1268	493.1346	−0.813	331.0822
Phenolic acids [M-H]^−^	Caffeoylquinic acid	12.3	C_16_H_18_O_9_	354.0968	353.089	5.083	191.0562; 179.034
	4-Caffeoylshikimic acid	15.3	C_16_H_16_O_8_	336.085	335.0772	2.975	179.0347; 161.0319; 135.0464
Flavones [M-H]^−^	6,8-di-C-hexosyl apigenin (vicenin-2)	14.5	C_27_H_30_O_15_	594.1581	593.1503	0.168	383.0763; 353.0662
	6-C-glycosyl luteolin (isoorientin)	16.9	C_21_H_20_O_11_	448.1002	447.0924	−1.785	447.0924; 357.0610; 327.0505
	6-C-glycosyl luteolin (isoorientin)	17.4	C_21_H_20_O_11_	448.1007	447.0929	−0.669	447.0929; 357.0617; 327.0499
	6-C-glycosyl luteolin (isoorientin)	17.6	C_21_H_20_O_11_	448.0988	447.091	−4.910	447.0910; 357.0588; 327.0488
	6-C-glycosyl apigenin (isovitexin)	19.0	C_21_H_20_O_10_	432.1053	431.0975	−1.620	431.0975; 311.0557; 283.0602; 341.0676
	Rhamnocitrin	33.2	C_16_H_12_O_6_	300.0633	299.0555	1.000	299; 284; 256; 227
Flavonoids [M-H]^−^	Taxifolin 3-*O*-glucoside	15.9	C_21_H_22_O_12_	466.1103	465.1025	−1,716	285.0409; 151.0035; 125.0728
	Rutin	19.0	C_27_H_30_O_16_	610.1513	609.1435	−2.786	255.0312; 271.0206. 301.0328
Flavanone [M-H]^−^	Eriodictyol 7-*O*-glucoside I	18.4	C_21_H_22_O_11_	450.1157	449.1079	−0.666	269.0441; 259.0616
Flavonols [M-H]^−^	Scoparin	20.1	C_22_H_22_O_11_	462.1160	461.1082	0.000	341.0667; 371.0776; 298.0483
	Scoparin	20.4	C_22_H_22_O_11_	462.1155	461.1077	0.000	341.0667; 371.0895; 298.0483

**Table 2:** The antioxidant capacity of the optimized açaí meal extract as determined by the total phenolic content (TPC), ferric-reducing antioxidant power (FRAP), and scavenging of ABTS radical cation (ABTS^•+^), and ROS assays [peroxyl radical (ROO^•^), superoxide anion (O_2_^•−^), and hypochlorous acid (HOCl)].

	Antioxidant Activity				
TPC (mg GAE∙g^−1^)	FRAP (µmol FS∙g^−1^)	ABTS^•+^ (µmol TE∙g^−1^)	ROO^∙^ (µmol TE∙g^−1^)	O_2_^−^ (EC_50_ µg∙mL^−1^)	HOCl (EC_50_ µg∙mL^−1^)
88.4 ± 0.4	986.0 ± 22.0	820.0 ± 36.4	975.7 ± 69.0	37.1 ± 1.9	4.2 ± 0.7

GAE—gallic acid equivalents; FS—ferrous sulfate; TE—Trolox equivalent. Data were expressed on a dry basis (mass of lyophilized açaí meal extract). The final values are the averages of the triplicates ± standard deviation.

**Table 3:** The Minimum Inhibitory Concentration (MIC) and Minimum Bactericidal Concentration (MBC) of the lyophilized optimized açaí meal extract.

Strain	Gram Staining	MIC/MBC (µg∙mL^−1^)
*Staphylococcus aureus* (ATCC 25923)	+	1250/1250
*S. aureus* (MRSA, ATCC 33591)	+	2500/2500
*Pseudomonas aeruginosa* (ATCC 27853)	−	2500/2500

## Data Availability

The data that support the finding of this study are available from the corresponding authors upon reasonable request.

## References

[1] Leal C., Gouvinhas I., Santos R.A., Rosa E., Silva A.M., Saavedra M.J., Barros A.I. (2020). Potential application of grape (*Vitis vinifera* L.) stem extracts in the cosmetic and pharmaceutical industries: Valorization of a by-product. Ind. Crop. Prod..

[2] Chang S.K., Alasalvar C., Shahidi F. (2018). Superfruits: Phytochemicals, antioxidant efficacies, and health effects—A comprehensive review. Crit. Rev. Food Sci. Nutr..

[3] Yamaguchi K.K.D.L., Pereira L.F.R., Lamarão C.V., Lima E.S., da Veiga-Junior V.F. (2015). Amazon acai: Chemistry and biological activities: A review. Food Chem..

[4] IBGE (2021). The Brazilian Institute of Geography and Statistics. Production of Plant Extraction and Forestry. Vegetable Extraction: Table 1—Amount Produced and the Production Value of Brazil, Great Regions and Federal Units, According to the Extractive Products. https://www.ibge.gov.br/estatisticas/economicas/agricultura-e-pecuaria/9105-producao-da-extracao-vegetal-e-da-silvicultura.html?=&t=resultados.

[5] Oliveira M.S.P., Schwartz G. (2018). oleraceae. Exotic Fruits.

[6] Kang J., Xie C.H., Li Z.M., Nagarajan S., Schauss A.G., Wu T., Wu X.L. (2011). Flavonoids from acai (*Euterpe oleraceae* Mart.) pulp and their antioxidant and anti-inflammatory activities. Food Chem..

[7] Embrapa (2005). Empresa Brasileira de Pesquisa Agropecuária, Sistemas de Produção 4—Açaí.

[8] Melo P.S., Selani M.M., Gonçalves R.H., Paulino J.d.O., Massarioli A.P., de Alencar S.M. (2021). Açaí seeds: An unexplored agro-industrial residue as a potential source of lipids, fibers, and antioxidant phenolic compounds. Ind. Crop. Prod..

[9] Yi C., Shi J., Kramer J., Xue S., Jiang Y., Zhang M., Ma Y., Pohorly J. (2009). Fatty acid composition and phenolic antioxidants of winemaking pomace powder. Food Chem..

[10] Hogan S., Chung H., Zhang L., Li J., Lee Y., Dai Y., Zhou K. (2010). Antiproliferative and antioxidant properties of anthocyanin-rich extract from açai. Food Chem..

[11] Schreckinger M.E., Lotton J., Lila M.A., De Mejia E.G. (2010). Berries from South America: A Comprehensive Review on Chemistry, Health Potential, and Commercialization. J. Med. Food.

[12] Rufino M.D.S.M., Pérez-Jiménez J., Arranz S., Alves R.E., de Brito E.S., Oliveira M.S., Saura-Calixto F. (2011). Açaí (*Euterpe oleraceaee*) ‘BRS Pará’: A tropical fruit source of antioxidant dietary fiber and high antioxidant capacity oil. Food Res. Int..

[13] Wong D.Y.S., Musgrave I.F., Harvey B.S., Smid S.D. (2013). Açaí (*Euterpe oleraceaee* Mart.) berry extract exerts neuroprotective effects against β-amyloid exposure *in vitro*. Neurosci. Lett..

[14] Bonomo L.D.F., Silva D.N., Boasquivis P.F., Paiva F.A., Guerra J.F.D.C., Martins T.A.F., Torres G.D.J., de Paula I.T.B.R., Caneschi W.L., Jacolot P. (2014). Açaí (*Euterpe oleraceae* Mart.) Modulates Oxidative Stress Resistance in Caenorhabditis elegans by Direct and Indirect Mechanisms. PLoS ONE.

[15] Ferreira D.S., Gomes A.L., Da Silva M.G., Alves A.B., Agnol W.H.D., Ferrari R.A., Carvalho P.R.N., Pacheco M.T.B. (2016). Antioxidant Capacity and Chemical Characterization of Açaí (*Euterpe oleraceae* Mart.) Fruit Fractions. Food Sci. Technol..

[16] Linan L.Z., Cidreira A.C.M., da Rocha C.Q., de Menezes F.F., de Moraes Rocha G.J., Paiva A.E.M. (2021). Utilization of Acai Berry Residual Biomass for Extraction of Lignocellulosic Byproducts. J. Bioresour. Bioprod..

[17] Rossetto R., Maciel G.M., Bortolini D.G., Ribeiro V.R., Haminiuk C.W.I. (2020). Acai pulp and seeds as emerging sources of phenolic compounds for enrichment of residual yeasts (*Saccharomyces cerevisiae*) through biosorption process. LWT.

[18] Tyug T.S., Prasad K.N., Ismail A. (2010). Antioxidant capacity, phenolics and isoflavones in soybean by-products. Food Chem..

[19] Zamindar N., Bashash M., Khorshidi F., Serjouie A., Shirvani M.A., Abbasi H., Sedaghatdoost A. (2017). Antioxidant efficacy of soybean cake extracts in soy oil protection. J. Food Sci. Technol..

[20] Halliwell B., Aeschbach R., Löliger J., Aruoma O. (1995). The characterization of antioxidants. Food Chem. Toxicol..

[21] Gomes A., Fernandes E., Silva A.M., Santos C.M., Pinto D.C., Cavaleiro J.A., Lima J.L. (2007). 2-Styrylchromones: Novel strong scavengers of reactive oxygen and nitrogen species. Bioorganic Med. Chem..

[22] Li H., Christman L.M., Li R., Gu L. (2020). Synergic interactions between polyphenols and gut microbiota in mitigating inflammatory bowel diseases. Food Funct..

[23] Romão M.H., de Bem G., Santos I.B., Soares R.D.A., Ognibene D., de Moura R.S., da Costa C.A., Resende C. (2019). Açaí (*Euterpe oleraceae* Mart.) seed extract protects against hepatic steatosis and fibrosis in high-fat diet-fed mice: Role of local renin-angiotensin system, oxidative stress and inflammation. J. Funct. Foods.

[24] Martinez R.M., Guimarães D.D.A.B., Berniz C.R., de Abreu J.P., da Rocha A.P.M., de Moura R.S., Resende A.C., Teodoro A.J. (2018). Açai (*Euterpe oleraceae* Mart.) Seed Extract Induces Cell Cycle Arrest and Apoptosis in Human Lung Carcinoma Cells. Foods.

[25] Wedick N.M., Pan A., Cassidy A., Rimm E.B., Sampson L., Rosner B., Willett W., Hu F.B., Sun Q., van Dam R.M. (2012). Dietary flavonoid intakes and risk of type 2 diabetes in US men and women. Am. J. Clin. Nutr..

[26] Circu M.L., Aw T.Y. (2010). Reactive oxygen species, cellular redox systems, and apoptosis. Free Radic. Biol. Med..

[27] Carpes S.T., Pereira D., De Moura C., Dos Reis A.S., Da Silva L.D., Oldoni T.L.C., Almeida J.F., Plata-Oviedo M.V.S. (2020). Lyophilized and microencapsulated extracts of grape pomace from winemaking industry to prevent lipid oxidation in chicken pâté. Braz. J. Food Technol..

[28] Silva A.P.D.S., Rosalen P.L., de Camargo A.C., Lazarini J.G., Rocha G., Shahidi F., Franchin M., de Alencar S.M. (2021). Inajá oil processing by-product: A novel source of bioactive catechins and procyanidins from a Brazilian native fruit. Food Res. Int..

[29] Singleton V.L., Orthofer R., Lamuela-Raventós R.M. (1999). Analysis of total phenols and other oxidation substrates and antioxidants by means of folin-ciocalteu reagent. Meth. Enzymol..

[30] Melo P.S., Massarioli A.P., Denny C., dos Santos L.F., Franchin M., Pereira G.E., Vieira T.M.F.D.S., Rosalen P.L., de Alencar S.M. (2015). Winery by-products: Extraction optimization, phenolic composition and cytotoxic evaluation to act as a new source of scavenging of reactive oxygen species. Food Chem..

[31] (2008). Clinical and Laboratory Standards Institute. Reference Method for Broth Dilution Antifungal Susceptibility Testing of Yeasts.

[32] (2012). Reference Method for Broth Dilution antibacterial Susceptibility Testing of Bacterial.

[33] Garzón G.A., Narváez-Cuenca C.-E., Vincken J.-P., Gruppen H. (2017). Polyphenolic composition and antioxidant activity of açai (*Euterpe oleraceae* Mart.) from Colombia. Food Chem..

[34] Fernandes I., Faria A., Azevedo J., Soares S., Calhau C., De Freitas V., Mateus N. (2010). Influence of Anthocyanins, Derivative Pigments and Other Catechol and Pyrogallol-Type Phenolics on Breast Cancer Cell Proliferation. J. Agric. Food Chem..

[35] Tan C., Kong Y., Tong Y., Deng H., Wang M., Zhao Y., Wan M., Lin S., Liu X., Meng X. (2021). Anti-apoptotic effects of high hydrostatic pressure treated cyanidin-3-glucoside and blueberry pectin complexes on lipopolysaccharide-induced inflammation in Caco-2 cells. J. Funct. Foods.

[36] Ma M.-M., Li Y., Liu X.-Y., Zhu W.-W., Ren X., Kong G.-Q., Huang X., Wang L.-P., Luo L.-Q., Wang X.-Z. (2015). Cyanidin-3-*O*-glucoside Ameliorates Lipopolysaccharide-Induced Injury Both In Vivo and In Vitro Suppression of NF-κB and MAPK Pathways. Inflammation.

[37] Li Z.-H., Guo H., Xu W.-B., Ge J., Li X., Alimu M., He D.-J. (2016). Rapid Identification of Flavonoid Constituents Directly from PTP1B Inhibitive Extract of Raspberry (*Rubus idaeus* L.) Leaves by HPLC–ESI–QTOF–MS-MS. J. Chromatogr. Sci..

[38] de Rosso V., Hillebrand S., Montilla E.C., Bobbio F.O., Winterhalter P., Mercadante A.Z. (2008). Determination of anthocyanins from acerola (*Malpighia emarginata* DC.) and açai (*Euterpe oleraceae* Mart.) by HPLC–PDA–MS/MS. J. Food Compos. Anal..

[39] Zhang Y., Seeram N.P., Lee R., Feng L., Heber D. (2008). Isolation and Identification of Strawberry Phenolics with Antioxidant and Human Cancer Cell Antiproliferative Properties. J. Agric. Food Chem..

[40] Yoshimura Y., Zaima N., Moriyama T., Kawamura Y. (2012). Different Localization Patterns of Anthocyanin Species in the Pericarp of Black Rice Revealed by Imaging Mass Spectrometry. PLoS ONE.

[41] Chen S., Zhou H., Zhang G., Meng J., Deng K., Zhou W., Wang H., Wang Z., Hu N., Suo Y. (2019). Anthocyanins from *Lycium ruthenicum* Murr. Ameliorated d-galactose-inducedmemory impairment, oxidative stress, and neuroinfammation in adult rats. J. Agric. Food Chem..

[42] Jugran A.K., Rawat S., Devkota H.P., Bhatt I.D., Rawal R.S. (2022). Diabetes andplant-derived natural products: From ethnopharmacological approaches to theirpotential for modern drug discovery and development. Phytoth. Res..

[43] Wei J., Zhang G., Zhang X., Xu D., Gao J., Fan J., Zhou Z. (2017). Anthocyanins from Black Chokeberry (*Aroniamelanocarpa* Elliot) Delayed Aging-Related Degenerative Changes of Brain. J. Agric. Food Chem..

[44] Liu F., Zhao F., Wang W., Sang J., Jia L., Li L., Lu F. (2020). Cyanidin-3-*O*-glucoside inhibits Aβ40 fibrillogenesis, disintegrates preformed fibrils, and reduces amyloid cytotoxicity. Food Funct..

[45] Li L., Zhou P., Wang Y., Pan Y., Chen M., Tian Y., Zhou H., Yang B., Meng H., Zheng J. (2022). Antimicrobial activity of cyanidin-3-*O*-glucoside–lauric acid ester against *Staphylococcus aureus* and *Escherichia coli*. Food Chem..

[46] Soares M.J., Sampaio G.R., Guizellini G.M., Figueira M.S., Pinaffi A.C.D.C., Freitas R.A.M.S., Shahidi F., de Camargo A.C., Torres E.A.F.D.S. (2020). Regular and decaffeinated espresso coffee capsules: Unravelling the bioaccessibility of phenolic compounds and their antioxidant properties in milk model system upon in vitro digestion. LWT.

[47] Naveed M., Hejazi V., Abbas M., Kamboh A.A., Khan G.J., Shumzaid M., Ahmad F., Babazadeh D., Xia F.F., Modarresi-Ghazani F. (2018). Chlorogenic acid (CGA): A pharmacological review and call for further research. Biomed. Pharmacother..

[48] Zhang Y., Xiong H., Xu X., Xue X., Liu M., Xu S., Liu H., Gao Y., Zhang H., Li X. (2018). Compounds Identification in Semen Cuscutae by Ultra-High-Performance Liquid Chromatography (UPLCs) Coupled to Electrospray Ionization Mass Spectrometry. Molecules.

[49] Kolniak-Ostek J., Oszmiański J. (2015). Characterization of phenolic compounds in different anatomical pear (*Pyrus communis* L.) parts by ultra-performance liquid chromatography photodiode detector-quadrupole/time of flight-mass spectrometry (UPLC-PDA-Q/TOF-MS). Int. J. Mass Spectrom..

[50] Brunschwig C., Leba L.-J., Saout M., Martial K., Bereau D., Robinson J.-C. (2016). Chemical Composition and Antioxidant Activity of *Euterpe oleraceae* Roots and Leaflets. Int. J. Mol. Sci..

[51] Zhong L., Lin Y., Wang C., Niu B., Xu Y., Zhao G., Zhao J. (2022). Chemical Profile, Antimicrobial and Antioxidant Activity Assessment of the Crude Extract and Its Main Flavonoids from Tartary Buckwheat Sprouts. Molecules.

[52] Ferreres F., Silva B.M., Andrade P.B., Seabra R.M., Ferreira M.A. (2003). Approach to the study of C-glycosyl flavones by ion trap HPLC-PAD-ESI/MS/MS: Application to seeds of quince (*Cydonia oblonga*). Phytochem. Anal..

[53] Kim M.K., Yun K.J., Lim D.H., Kim J., Jang Y.P. (2016). Anti-Inflammatory Properties of Flavone di-C-Glycosides as Active Principles of Camellia Mistletoe, *Korthalsella japonica*. Biomol. Ther..

[54] Odontuya G., Hoult J.R.S., Houghton P.J. (2005). Structure-activity relationship for antiinflammatory effect of luteolin and its derived glycosides. Phytother. Res..

[55] Francisco V., Figueirinha A., Costa G., Liberal J., Lopes M.C., García-Rodríguez C., Geraldes C.F., Cruz M.T., Batista M.T. (2014). Chemical characterization and anti-inflammatory activity of luteolin glycosides isolated from lemongrass. J. Funct. Foods.

[56] Jang D., Jung Y.S., Kim M.-S., Oh S.E., Nam T.G., Kim D.-O. (2019). Developing and Validating a Method for Separating Flavonoid Isomers in Common Buckwheat Sprouts Using HPLC-PDA. Foods.

[57] Chen G.-L., Mutie F.M., Xu Y.-B., Saleri F.D., Hu G.-W., Guo M.-Q. (2020). Antioxidant, Anti-inflammatory Activities and Polyphenol Profile of *Rhamnus prinoides*. Pharmaceuticals.

[58] Martini N., Katerere D., Eloff J. (2004). Biological activity of five antibacterial flavonoids from *Combretum erythrophyllum* (Combretaceae). J. Ethnopharmacol..

[59] Bodakhe S.H., Ram A., Verma S., Pandey D.P. (2012). Anticataract activity of rhamnocitrin isolated from *Bauhinia variegata* stem bark. Orient. Pharm. Exp. Med..

[60] Cuoco G., Mathe C., Vieillescazes C. (2014). Liquid chromatographic analysis of flavonol compounds in green fruits of three *Rhamnus* species used in Stil de grain. Microchem. J..

[61] Kim B.-R., Paudel S.B., Han A.-R., Park J., Kil Y.-S., Choi H., Jeon Y.G., Park K.Y., Kang S.-Y., Jin C.H. (2021). Metabolite Profiling and Dipeptidyl Peptidase IV Inhibitory Activity of *Coreopsis* Cultivars in Different Mutations. Plants.

[62] Chen M., Zhang X., Wang H., Lin B., Wang S., Hu G. (2015). Determination of Rutin in Rat Plasma by Ultra Performance Liquid Chromatography Tandem Mass Spectrometry and Application to Pharmacokinetic Study. J. Chromatogr. Sci..

[63] Ben Sghaier M., Pagano A., Mousslim M., Ammari Y., Kovacic H., Luis J. (2016). Rutin inhibits proliferation, attenuates superoxide production and decreases adhesion and migration of human cancerous cells. Biomed. Pharmacother..

[64] Zhou C., Xie Z., Lei Z., Huang Y., Wei G. (2018). Simultaneous identification and determination of flavonoids in *Dendrobium officinale*. Chem. Central J..

[65] Dias A.L.S., Rozet E., Larondelle Y., Hubert P., Rogez H., Quetin-Leclercq J. (2013). Development and validation of an UHPLC-LTQ-Orbitrap MS method for non-anthocyanin flavonoids quantification in *Euterpe oleraceae* juice. Anal. Bioanal. Chem..

[66] Ho K.-V., Lei Z., Sumner L.W., Coggeshall M.V., Hsieh H.-Y., Stewart G.C., Lin C.-H. (2018). Identifying Antibacterial Compounds in Black Walnuts (*Juglans nigra*) Using a Metabolomics Approach. Metabolites.

[67] Lee J.K. (2011). Anti-inflammatory effects of eriodictyol in lipopolysaccharidestimulated raw 264.7 murine macrophages. Arch. Pharmacal Res..

[68] Mulabagal V., Calderón A.I. (2012). Liquid chromatography/mass spectrometry based fingerprinting analysis and mass profiling of *Euterpe oleraceae* (açaí) dietary supplement raw materials. Food Chem..

[69] Prior R.L., Cao G., Martin A., Sofic E., McEwen J., O’Brien C., Lischner N., Ehlenfeldt M., Kalt W., Krewer A.G. (1998). Antioxidant Capacity As Influenced by Total Phenolic and Anthocyanin Content, Maturity, and Variety of *Vaccinium* Species. J. Agric. Food Chem..

[70] de Camargo A.C., Regitano-D’Arce M.A.B., Biasoto A.C.T., Shahidi F. (2014). Low Molecular Weight Phenolics of Grape Juice and Winemaking Byproducts: Antioxidant Activities and Inhibition of Oxidation of Human Low-Density Lipoprotein Cholesterol and DNA Strand Breakage. J. Agric. Food Chem..

[71] Ayoub M., de Camargo A.C., Shahidi F. (2016). Antioxidants and bioactivities of free, esterified and insoluble-bound phenolics from berry seed meals. Food Chem..

[72] Butkevičiūtė A., Urbštaitė R., Liaudanskas M., Obelevičius K., Janulis V. (2022). Phenolic Content and Antioxidant Activity in Fruit of the Genus *Rosa* L.. Antioxidants.

[73] Kang J., Thakali K.M., Xie C., Kondo M., Tong Y., Ou B., Jensen G., Medina M.B., Schauss A.G., Wu X. (2012). Bioactivities of açaí (*Euterpe precatoria* Mart.) fruit pulp, superior antioxidant and anti-inflammatory properties to *Euterpe oleraceae* Mart. Food Chem..

[74] Batista C.D.C.R., de Oliveira M.S., Araújo M.E., Rodrigues A.M.C., Botelho J.R.S., da Silva Souza Filho A.P., Machado N., Carvalho Junior R.N. (2016). Supercritical CO 2 extraction of açaí (*Euterpe oleraceae*) berry oil: Global yield, fatty acids, allelopathic activities, and determination of phenolic and anthocyanins total compounds in the residual pulp. J. Supercrit. Fluids.

[75] Sette P., Fernandez A., Soria J., Rodriguez R., Salvatori D., Mazza G. (2020). Integral valorization of fruit waste from wine and cider industries. J. Clean. Prod..

[76] Kumar M., Dahuja A., Tiwari S., Punia S., Tak Y., Amarowicz R., Bhoite A.G., Singh S., Joshi S., Panesar P.S. (2021). Recent trends in extraction of plant bioactives using green technologies: A review. Food Chem..

[77] Tremocoldi M.A., Rosalen P.L., Franchin M., Massarioli A.P., Denny C., Daiuto E.R., Paschoal J.A.R., Melo P.S., de Alencar S.M. (2018). Exploration of avocado by-products as natural sources of bioactive compounds. PLoS ONE.

[78] de Moura C., dos Reis A.S., da Silva L.D., de Lima V.A., Oldoni T.L.C., Pereira C., Carpes S.T. (2018). Optimization of phenolic compounds extraction with antioxidant activity from acai, blueberry and goji berry using response surface methodology. Emir. J. Food Agric..

[79] Silva F., De Miranda D., Carnier M., Maza P., Boldarine V., Rischiteli A.S., Avila F., Pontes L., Hachul A., Neto N. (2021). Low dose of Juçara pulp (*Euterpe edulis* Mart.) minimizes the colon inflammatory milieu promoted by hypercaloric and hyperlipidic diet in mice. J. Funct. Foods.

[80] Morais D.R., Rotta E.M., Sargi S.C., Schmidt E.M., Bonafe E.G., Eberlin M.N., Sawaya A.C., Visentainer J.V. (2015). Antioxidant activity, phenolics and UPLC–ESI(–)–MS of extracts from different tropical fruits parts and processed peels. Food Res. Int..

[81] Tiveron A.P., Melo P.S., Bergamaschi K.B., Vieira T.M.F.D.S., Regitano-D’Arce M.A.B., De Alencar S.M. (2012). Antioxidant Activity of Brazilian Vegetables and Its Relation with Phenolic Composition. Int. J. Mol. Sci..

[82] Infante J., Rosalen P.L., Lazarini J.G., Franchin M., De Alencar S.M. (2016). Antioxidant and Anti-Inflammatory Activities of Unexplored Brazilian Native Fruits. PLoS ONE.

[83] Lazarini J.G., Franchin M., Soares J.C., Nani B.D., Massarioli A.P., De Alencar S.M., Rosalen P.L. (2020). Anti-inflammatory and antioxidant potential, in vivo toxicity, and polyphenolic composition of *Eugenia selloi* B.D.Jacks. (pitangatuba), a Brazilian native fruit. PLoS ONE.

[84] Soares J.C., Rosalen P.L., Lazarini J.G., Massarioli A.P., da Silva C.F., Nani B.D., Franchin M., de Alencar S.M. (2019). Comprehensive characterization of bioactive phenols from new Brazilian superfruits by LC-ESI-QTOF-MS, and their ROS and RNS scavenging effects and anti-inflammatory activity. Food Chem..

[85] da Silveira T.F.F., de Souza T.C.L., Carvalho A.V., Ribeiro A.B., Kuhnle G.G., Godoy H.T. (2017). White açaí juice (*Euterpe oleraceae*): Phenolic composition by LC-ESI-MS/MS, antioxidant capacity and inhibition effect on the formation of colorectal cancer related compounds. J. Funct. Foods.

[86] Rodrigues E., Mariutti L.R.B., Mercadante A.Z. (2013). Carotenoids and Phenolic Compounds from *Solanum sessiliflorum*, an Unexploited Amazonian Fruit, and Their Scavenging Capacities against Reactive Oxygen and Nitrogen Species. J. Agric. Food Chem..

[87] Sprenger L.K., Giese E.G., Dos Santos J.N., Molento M.B. (2016). In vitro antibacterial effect of *Euterpe oleraceae* Mart. and Theobroma grandiflorum hydroalcoholic extracts. Arch. Vet. Sci..

[88] Dias-Souza M.V., dos Santos R.M., Cerávolo I.P., Cosenza G., Marçal P.H.F., Figueiredo F.J.B. (2018). *Euterpe oleraceae* pulp extract: Chemical analyses, antibiofilm activity against Staphylococcus aureus, cytotoxicity and interference on the activity of antimicrobial drugs. Microb. Pathog..

[89] Golovinskaia O., Wang C.-K. (2021). Review of Functional and Pharmacological Activities of Berries. Molecules.

[90] Zhang H., Tsao R. (2016). Dietary polyphenols, oxidative stress and antioxidant and anti-inflammatory effects. Curr. Opin. Food Sci..

[91] Xie C., Kang J., Li Z., Schauss A., Badger T.M., Nagarajan S., Wu T., Wu X. (2012). The açaí flavonoid velutin is a potent anti-inflammatory agent: Blockade of LPS-mediated TNF-α and IL-6 production through inhibiting NF-κB activation and MAPK pathway. J. Nutr. Biochem..

[92] Brito C., Stavroullakis A., Oliveira T., Prakki A. (2017). Cytotoxicity and potential anti-inflammatory activity of velutin on RAW 264.7 cell line differentiation: Implications in periodontal bone loss. Arch. Oral Biol..

[93] Daglia M. (2012). Polyphenols as antimicrobial agents. Curr. Opin. Biotechnol..

[94] Nile S.H., Park S.W. (2014). Edible berries: Bioactive components and their effect on human health. Nutrition.

[95] Xue J., Davidson P.M., Zhong Q. (2013). Thymol Nanoemulsified by Whey Protein-Maltodextrin Conjugates: The Enhanced Emulsifying Capacity and Antilisterial Properties in Milk by Propylene Glycol. J. Agric. Food Chem..

[96] Ultee A., Bennik M.H.J., Moezelaar R. (2002). The Phenolic Hydroxyl Group of Carvacrol Is Essential for Action against the Food-Borne Pathogen *Bacillus cereus*. Appl. Environ. Microbiol..

[97] Newman D.J., Cragg G.M. (2020). Natural products as sources of new drugs over the nearly four decades from 01/1981 to 09/2019. J. Nat. Prod..

[98] Freires I.A., Denny C., Benso B., De Alencar S.M., Rosalen P.L. (2015). Antibacterial Activity of Essential Oils and Their Isolated Constituents against Cariogenic Bacteria: A Systematic Review. Molecules.

